# Primary glioblastoma mimicking brain metastasis in ALK-positive lung adenocarcinoma: a case report and literature review

**DOI:** 10.3389/fonc.2026.1837876

**Published:** 2026-05-15

**Authors:** Mengyuan Li, Hanghuang Jin, Jianhua Luo, Cheng Zheng

**Affiliations:** 1 Department of Pulmonary and Critical Care Medicine, Taizhou Municipal Hospital (Taizhou University Affiliated Municipal Hospital), School of Medicine, Taizhou, China; 2 Department of Neurosurgery, Taizhou Municipal Hospital (Taizhou University Affiliated Municipal Hospital), School of Medicine, Taizhou, China; 3 Department of Critical Care Medicine, Taizhou Municipal Hospital (Taizhou University Affiliated Municipal Hospital), School of Medicine, Taizhou, China; 4 Taizhou Key Laboratory of Sepsis, Taizhou Municipal Hospital, Taizhou, China; 5 Taizhou Key Laboratory of Infection and Tumor Immunology, Taizhou Municipal Hospital, Taizhou, China

**Keywords:** brain metastasis, case report, double primary cancers, glioblastoma, lung cancer, misdiagnosis

## Abstract

In patients with a known diagnosis of lung cancer, a new intracranial space-occupying lesion is most frequently interpreted as a brain metastasis. However, distinguishing it from an independent double primary brain tumor is critical, as treatment strategies and prognoses differ substantially. We report a rare case of primary glioblastoma occurring approximately two years after surgery for anaplastic lymphoma kinase (ALK)-positive lung adenocarcinoma. A 63-year-old female underwent radical resection for stage IA lung adenocarcinoma (ALK-positive) and received adjuvant ensartinib. Two years postoperatively, brain magnetic resonance imaging (MRI) revealed a solitary lesion in the right centrum semiovale, which was clinically misdiagnosed as a brain metastasis. The patient was subsequently treated with lorlatinib combined with stereotactic radiotherapy. Despite these interventions, she developed progressive left-sided limb weakness, and imaging demonstrated continuous lesion progression. Subsequent surgical resection and pathological examination confirmed primary glioblastoma, strongly supporting a diagnosis of metachronous double primary cancers (DPCs). The patient died 10 months after the glioblastoma diagnosis. This case highlights that during long-term follow-up of patients with malignancies, a new intracranial lesions may warrant consideration of a second primary cancer (SPC), particularly when the treatment response is not consistent with the expected biology of the original tumor. Timely pathological confirmation and multidisciplinary review may help reduce diagnostic delay and improve treatment selection.

## 1 Introduction

Lung cancer ranks first in both incidence and mortality among all malignancies worldwide and remains the leading cause of cancer-related deaths (1, 2). Among the various molecular subtypes of lung cancer, patients with lung adenocarcinoma harboring anaplastic lymphoma kinase (ALK) gene rearrangements have achieved significantly prolonged survival due to the clinical application of ALK-tyrosine kinase inhibitors (TKIs) (3–5). However, the brain is one of the most common sites of distant metastasis in lung cancer, and ALK-positive patients are at particularly high risk for brain metastases. Among patients with ALK-positive metastatic non-small cell lung cancer (mNSCLC), 25% to 37% present with brain metastases at initial diagnosis (3–5). Furthermore, among those without baseline brain metastases, the cumulative incidence of new brain metastases within five years of follow-up reaches 20% to 22% (6, 7). Thus, this patient population bears a substantial burden of brain metastases throughout the disease course. Consequently, when a new intracranial lesion is detected during follow-up in an ALK-positive lung cancer patient, clinical reasoning often becomes habitually anchored to the diagnosis of “brain metastasis”, leading to adjustments in TKI treatment strategies or the addition of local radiotherapy. However, a critical diagnostic pitfall that cannot be overlooked in clinical practice is the possibility that the patient may develop a primary brain tumor independent of the lung cancer. Double primary cancers (DPC) of lung cancer and glioma are extremely rare, with only seven cases reported in the literature to date (8–14); Notably, to our knowledge, no such case has been previously reported in the available literature. Moreover, brain metastases and primary brain tumors can both manifest as ring-enhancing lesions with peritumoral edema on imaging, making differentiation by conventional magnetic resonance imaging (MRI) exceedingly challenging (15, 16). This article reports a case of a patient with ALK-positive lung adenocarcinoma who developed a new glioblastoma postoperatively that was persistently misdiagnosed as a brain metastasis, leading to inappropriate targeted therapy and delay of standard treatment. This case serves as a cautionary reminder that in the era of targeted therapy, a lack of vigilance for second primary cancer (SPC) can lead to significant diagnostic and therapeutic delays.

## 2 Case presentation

**Figure 1:**
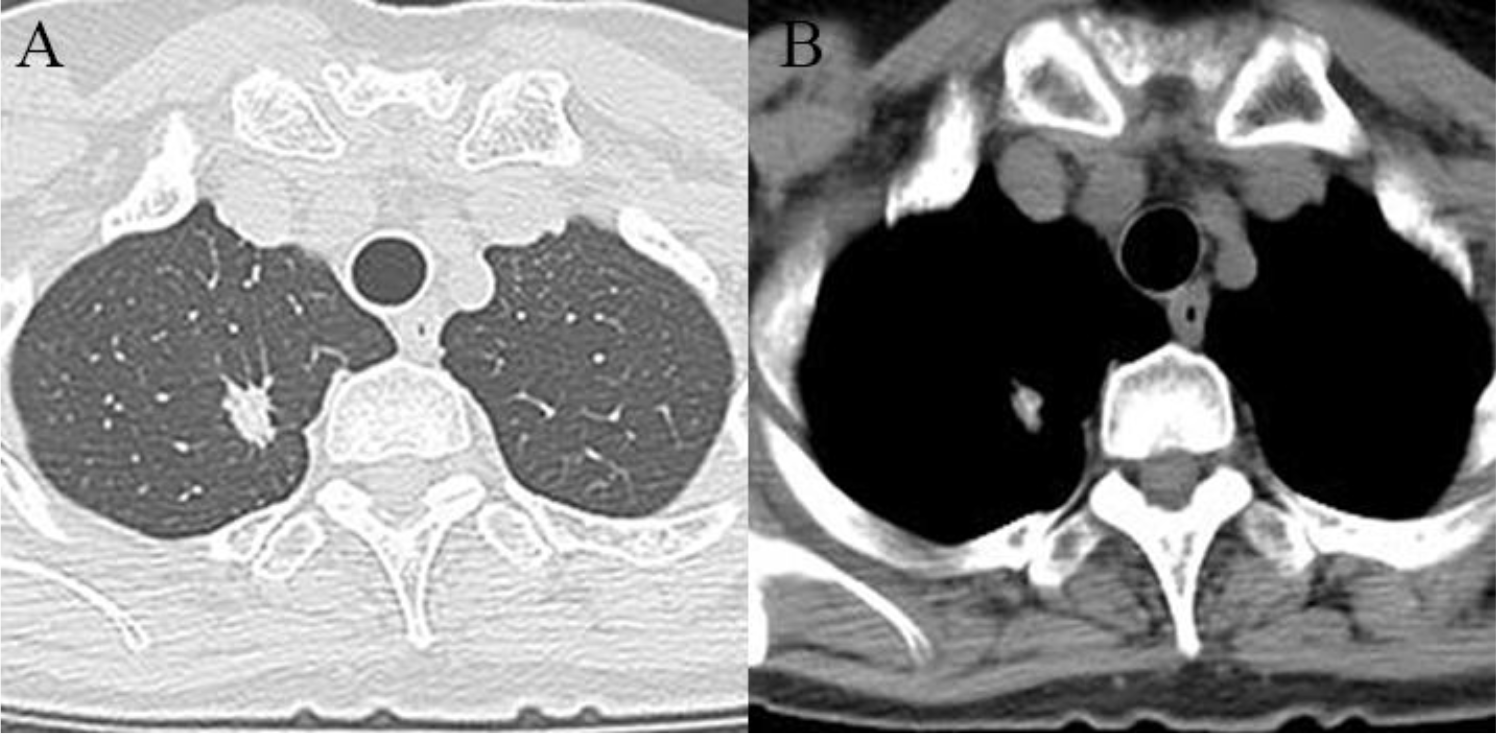
Chest CT scan on October 27, 2022. **(A)** Lung window; **(B)** Mediastinal window. showing an irregular solid nodule in the right upper lobe, 1.5cm × 0.8cm.

**Figure 2:**
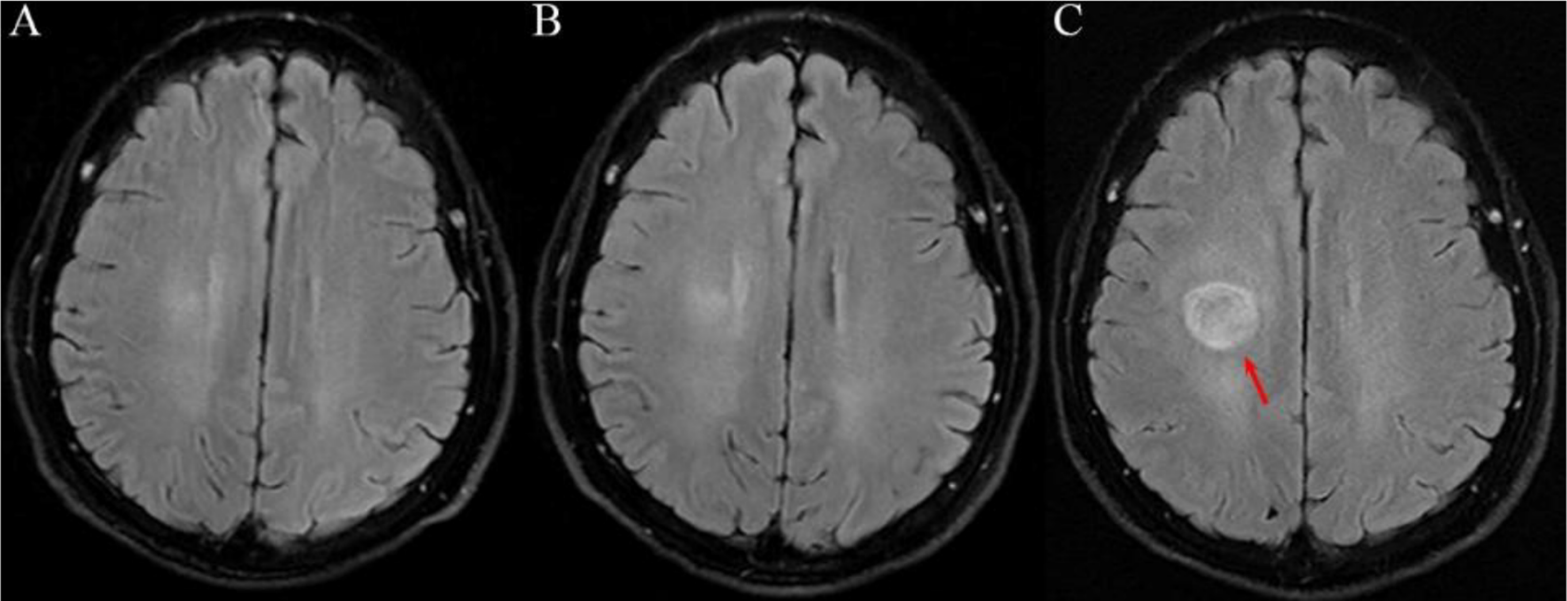
Serial brain MRI findings. **(A)** Preoperative MRI (November 14, 2022) showing no metastasis. **(B)** Follow-up MRI (April 12, 2024), negative. **(C)** MRI (November 6, 2024). T2 FLAIR image showing a new solitary lesion in the right centrum semiovale, 2.5 cm × 2.0 cm (arrow).

A 63-year-old female of Chinese ethnicity with no smoking history underwent a routine health examination on October 27, 2022. Chest computed tomography (CT) revealed an irregular solid nodule in the right upper lobe, measuring approximately 1.5 cm × 0.8 cm (**Figure 1**). She had no respiratory symptoms. Preoperative brain magnetic resonance imaging (MRI) on November 14, 2022, showed no evidence of intracranial metastasis (**Figure 2A**). On November 15, 2022, she underwent right upper lobectomy with lymph node dissection at an outside hospital. Postoperative pathological examination revealed poorly differentiated adenocarcinoma (predominantly micropapillary pattern, partially acinar pattern), with tumor dimensions of 1.5 cm × 1.3 cm × 1.1 cm. Spread through air spaces (STAS) was observed, with no lymphovascular or perineural invasion. All dissected lymph nodes were negative for malignancy. Next-generation sequencing (NGS) of the tumor tissue revealed an echinoderm microtubule-associated protein-like 4-ALK (EML4-ALK) fusion, confirming ALK-positive status. The patient was diagnosed with right lung adenocarcinoma (pT1bN0M0, stage IA, 8th edition). Although adjuvant ALK-TKI therapy is not the current standard of care for stage IA disease and was therefore used off-label in this case, the tumor exhibited several high-risk pathological features, including a predominant solid/micropapillary pattern, spread through air spaces (STAS), and poor differentiation. After thorough discussion with the patient regarding the potential risks and uncertain benefits, postoperative adjuvant targeted therapy with ensartinib (225 mg orally once daily) was initiated, and regular follow-up was recommended.

**Figure 3:**
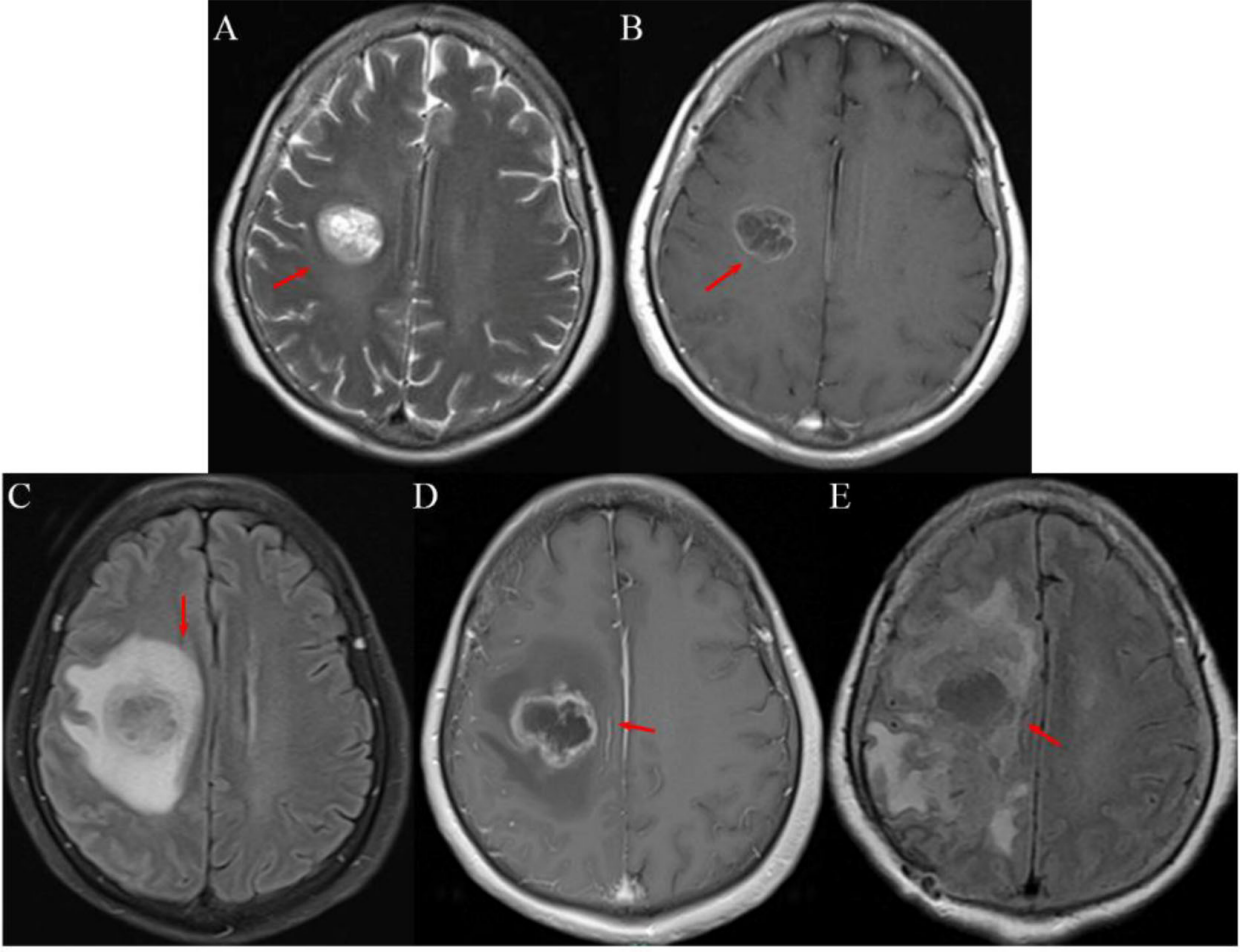
**(A, B)** MRI (November 26, 2024). **(A)** T2 TSE image showing the lesion with surrounding edema (arrow). **(B)** Contrast-enhanced T1-weighted image showing ring enhancement, with lesion measuring 2.6 cm × 2.2 cm (arrow). **(C, D)** MRI (January 21, 2025). **(C)** T2 FLAIR image showing further enlargement of the lesion with markedly increased peritumoral edema (arrow). **(D)** Contrast-enhanced T1-weighted image showing lesion measuring 2.7 cm × 2.5 cm with persistent ring enhancement (arrow). **(E)** MRI (September 1, 2025) T2 FLAIR image showing significant tumor progression with lesion measuring 5.0 cm × 8.0 cm after glioblastoma diagnosis (arrow).

Serial follow-up chest CT scans showed no evidence of local recurrence or new pulmonary lesions. Follow-up brain MRI on December 15, 2023, and April 12, 2024, both showed no intracranial metastasis (**Figure 2B**). On November 6, 2024, a routine follow-up brain MRI revealed a newly developed solitary lesion in the right centrum semiovale, measuring approximately 2.5 cm × 2.0 cm (**Figure 2C**). At that time, the patient reported no neurological symptoms. The lesion was clinically suspected to be a brain metastasis, and targeted therapy was switched to lorlatinib (100 mg orally once daily) on November 12, 2024. On November 24, 2024, the patient developed left-sided limb weakness (grade IV), gait instability, and blurred vision. Contrast-enhanced brain MRI on November 26, 2024, showed an enlarged lesion in the right centrum semiovale, measuring approximately 2.6 cm × 2.2 cm, with surrounding edema (**Figures 3A, B**). Given the progressive neurological deficits and MRI-confirmed local progression with poor response to lorlatinib, intracranial radiotherapy was indicated. Accordingly, stereotactic radiotherapy was performed without immediate biopsy, as it was regarded as the standard local treatment for presumed brain metastasis. From November 26 to 28, 2024, she underwent stereotactic radiotherapy (6mv-X, source-axis distance 100, DT 2700 cGy in 3 fractions over 3 days). However, her symptoms did not improve, and left-sided limb weakness progressively worsened from grade IV to grade 0, accompanied by mild headache and occasional vomiting. Follow-up contrast-enhanced brain MRI on January 21, 2025, revealed further enlargement of the lesion to approximately 2.7 cm × 2.5 cm (**Figures 3C, D**).

The patient was referred to Taizhou Municipal Hospital (Taizhou University Affiliated Municipal Hospital) for further management. Given the lesion’s progressive enlargement despite targeted therapy and radiotherapy, along with worsening neurological deficits, a multidisciplinary team (MDT) discussion was convened at our hospital. The consensus was that the lesion exhibited treatment resistance inconsistent with the expected behavior of a brain metastasis from ALK-positive lung cancer, and that pathological confirmation was necessary. The indications for surgical resection were: (1) treatment resistance necessitating pathological diagnosis; and (2) progressive neurological deficits requiring relief of mass effect. On February 7, 2025, the patient underwent craniotomy for right frontal lobe tumor resection.

**Figure 4:**
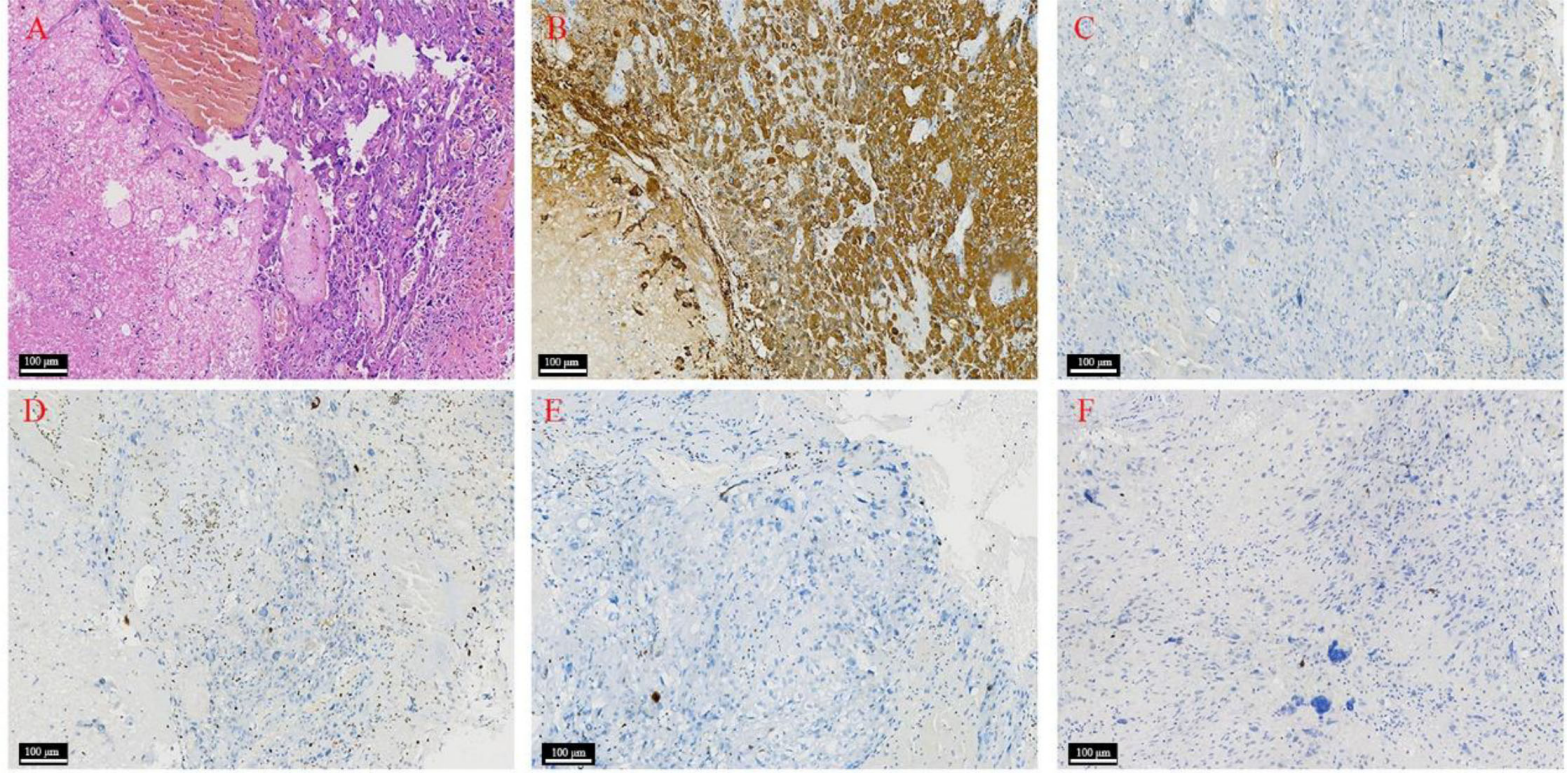
Pathological examination of the intracranial lesion. **(A)** Hematoxylin and eosin staining showing gemistocytic astrocytes and multinucleated atypical cells with prominent necrosis (scale bar: 100 μm). **(B)** GFAP positive (scale bar: 100 μm). **(C)** IDH-1 negative (scale bar: 100 μm). **(D)** Ki-67 proliferation index showing approximately 15% positivity in hot spots (scale bar: 100 μm). **(E)** Napsin A negative (scale bar: 100 μm). **(F)** TTF-1 negative (scale bar: 100 μm).

Postoperative pathological examination revealed a malignant glial neoplasm characterized by gemistocytic astrocytes and multinucleated atypical cells with prominent necrosis. Immunohistochemical staining showed the tumor cells were positive for GFAP and Vimentin, with partially strong positivity for P53. Stains for epithelial markers (NapsinA, TTF-1, CK7, AE1/AE3) and other lineage-specific markers (CK5/6, P40, CD56) were negative. IDH1 R132H immunohistochemistry was negative. The Ki-67 proliferation index was approximately 15% in hot spots (**Figure 4**). Molecular analysis revealed a TERT (telomerase reverse transcriptase) promoter C228T mutation; the C250T mutation was not detected. The histopathological and molecular features were consistent with a diagnosis of *glioblastoma, IDH-wildtype* (WHO grade 4).

**Figure 5:**
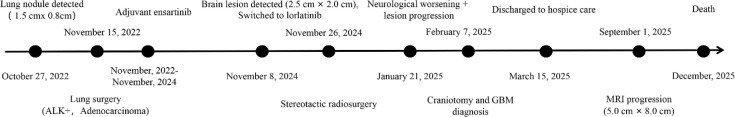
Treatment timeline.

Postoperatively, the patient’s left-sided limb weakness remained grade 0. During hospitalization, she developed a pulmonary infection, which improved with antibiotic therapy. Given her poor functional status and the family’s request for conservative supportive care, no postoperative adjuvant chemotherapy or radiotherapy was administered. She was discharged on March 15, 2025. Follow-up brain MRI on September 1, 2025, showed significant tumor progression (**Figure 3E**). The patient died in December 2025, 10 months after the diagnosis of glioblastoma. The patient’s clinical course is summarized in the treatment timeline (**Figure 5**).

## 3 Discussion

In the current era where targeted therapy has significantly prolonged the survival of patients with ALK-positive lung cancer (3–5), the differential diagnosis of SPC during long-term follow-up has become increasingly important. We report a rare case of a patient with ALK-positive lung adenocarcinoma who, in the absence of baseline brain metastases, developed a new intracranial lesion two years postoperatively, which was ultimately diagnosed as primary glioblastoma rather than brain metastasis. This rare double primary combination was persistently misdiagnosed in clinical practice, leading to the patient receiving ineffective ALK-TKI therapy and consequently delaying standard surgical resection, chemotherapy, and radiotherapy for glioblastoma. To the best of our knowledge, the double primary cancer combination of ALK-positive lung adenocarcinoma and glioblastoma has not been previously reported. This case reveals that in the era of long-term survival management, insufficient awareness of the differential diagnosis of SPC can lead to significant diagnostic and therapeutic delays.

To systematically analyze the clinical characteristics of DPC of lung cancer and glioma, We searched PubMed, Embase, Web of Science, and Google Scholar up to February 2026 and also screened non-English reports when sufficient bibliographic and case-level information was available. The retrieval formula was (lung cancer OR lung neoplasm OR pulmonary adenocarcinoma) AND (glioblastoma OR glioma OR astrocytoma). The search was limited to English-language articles with available full text. After searching by keywords and carefully reading the original articles, a total of seven articles containing information on seven patients were finally included (8–14). Combined with the present case of lung adenocarcinoma who developed a new intracranial lesion postoperatively, a total of eight patients were included in the pooled analysis (**Table 1**). Regarding tumor pathology and molecular characteristics, lung cancer subtypes were predominantly adenocarcinoma (6/8), followed by adenosquamous carcinoma (1/8) and small cell lung cancer (1/8). Driver gene mutations were detected in three cases, including one case each of ALK-positive (the present case), Kirsten rat sarcoma viral oncogene homolog (KRAS) G12C mutation, and epidermal growth factor receptor (EGFR) L858R mutation. Glioma subtypes included glioblastoma (6/8), malignant astrocytoma (1/8), and gliosarcoma (1/8). In terms of temporal distribution and imaging characteristics, synchronous occurrence of the two malignancies was observed in six cases, while metachronous occurrence was observed in two cases. Notably, three patients had concurrent brain metastases at the time of glioma diagnosis. Regarding imaging findings, intracranial lesions were presented as solitary in the majority of cases (7/8), with only one case presenting as multiple lesions. Analysis of diagnostic delays and their underlying causes revealed that clinical reasoning preferentially favored brain metastasis from lung cancer, leading to diagnostic delays in multiple cases, ranging from 3 to 51 months. Definitive diagnosis ultimately depended on lesion biopsy or surgical resection. Factors prompting pathological biopsy included: persistent treatment resistance, significant mass effect requiring emergency surgical decompression, or imaging features atypical for metastatic tumors.

**Table 1:** Summary of clinical characteristics in lung cancer patients with co-existing primary brain glioma.

Case	Age/Sex	Smoking history	Lung cancer pathology (genotype)	Temporal relationship	Key imaging features	Co-existing brain metastases?	Glioma pathology(genotype)	Misdiagnosis/Delay interval	Definitive diagnostic method	Trigger for biopsy/surgery	Outcome
Kishimoto (8)	54/M	40 cigs/day × 34 years	Adenocarcinoma (Not mentioned)	Synchronous	Single lesion, left lentiform nucleus (3 cm)	No	Malignant Astrocytoma (Genotype not mentioned)	3 months	Autopsy	No biopsy, treated as metastasis directly	Death
Bari(9)	75/F	2 packs/day	Small Cell Lung Cancer	Synchronous	Single,large mass in right frontal lobe (6 cm)	No	Glioblastoma (Genotype not mentioned)	No delay (synchronous surgery)	Craniotomy	Acute mass effect requiring decompression	Not specified
Nukaga(10)	66/F	Non-smoker	Adenocarcinoma (EGFR L858R+)	Metachronous (7 years later)	Solitary cerebellar lesion showing isolated rapid progression among multiple brain metastases	Yes	Glioblastoma (Genotype not mentioned)	13 months	Craniotomy	Isolated treatment resistance	Not specified
Qian(11)	60/F	30 pack-year	Adenocarcinoma (KRAS G12C+, PD-L1 >50%)	Synchronous	New right temporal lesion; other brain metastases present and responding to treatment	Yes	Glioblastoma (IDH-wildtype, MGMT methylated)	16 months	Craniotomy	Discordant treatment response with acute neurological symptoms	Hospice care
Shahsavari(12)	70/F	49 pack-year	Adenocarcinoma (Not mentioned)	Synchronous	Right temporal lesion; other brain metastases present	Yes	Glioblastoma (IDH-wildtype, MGMT unmethylated, p53+, ATRX+)	51 months	Stereotactic Biopsy	Persistent treatment resistance	Death 5 months after diagnosis
Carmicheal(13)	65/M	27.75 pack-year	Adenocarcinoma (Not mentioned)	Synchronous	Single cystic/solid lesion in right anterior temporal lobe (2.3 cm).	No	Glioblastoma (IDH-wildtype, MGMT unmethylated)	8 months	Craniotomy (partial resection)	Explosive progression post-SRS	Hospice care
Hijab(14)	76/F	Not mentioned	Adenosquamous Carcinoma (Driver gene negative, PD-L1 5%)	Synchronous	Multiple 3 lesions, involving corpus callosum and fornices (atypical distribution)	No	Glioblastoma (Genotype not mentioned)	Diagnosis confirmed before treatment	Stereotactic Biopsy	Atypical imaging distribution	Symptom improvement, stable intracranial disease
Present Case	63/F	Non-smoker	Adenocarcinoma (EML4-ALK+)	Metachronous (2 years)	Solitary lesion in right centrum semiovale (2.5 cm × 2.0 cm)	No	Glioblastoma (IDH-wildtype)	3 months	Craniotomy	Treatment resistance and progressive neurological deterioration	Death 10 months after GBM diagnosis

SRS, stereotactic radiosurgery; GBM, glioblastoma; EML4-ALK, echinoderm microtubule-associated protein-like 4-anaplastic lymphoma kinase; KRAS, Kirsten rat sarcoma viral oncogene homolog; EGFR, epidermal growth factor receptor; PD-L1, programmed death-ligand 1.

Several factors contributed to the misdiagnosis of brain metastasis in this patient. First, although the patient had postoperative stage IA lung adenocarcinoma, the pathological subtype was predominantly solid/micropapillary pattern with STAS and poor differentiation, all of which are high-risk factors for postoperative recurrence and metastasis in early-stage lung cancer (17–19). Concurrently, ALK fusion-positive lung cancer itself is a high-risk subtype for brain metastases, with the cumulative incidence of new brain metastases within five years of follow-up reaching as high as 20% to 22% (6, 7). Additionally, the patient had no intracranial metastases at baseline or during regular postoperative follow-up; this “clean” background paradoxically made the first-appearing solitary lesion more consistent with the clinical expectation of “first-episode brain metastasis”. At the imaging level, distinguishing between solitary brain metastasis and glioblastoma on conventional MRI has long been a significant clinical challenge (15). Both frequently present as solitary, ring-enhancing lesions with pronounced peritumoral edema, with highly overlapping imaging features (15). Although several studies have attempted to differentiate them using specific signs such as signal alteration in the adjacent cortex, peripheral rim sign, and the ratio of peritumoral edema to enhancing tumor, their diagnostic accuracy remains below 70% (20, 21). Moreover, these subtle differences are easily overlooked or ambiguously interpreted in routine clinical reading. Therefore, when a high-risk clinical background is combined with non-specific imaging findings, “brain metastasis” becomes a seemingly unquestionable diagnosis, directly leading to delayed recognition of the critical contradictory signal of treatment resistance. Of note, although recent deep learning-based radiomics models have shown promise in distinguishing glioblastoma from solitary brain metastases (22, 23), such tools face challenges including data heterogeneity and poor external validation, and have not yet been implemented in routine clinical practice (24, 25).

The critical turning point in the diagnostic process of this case lay in the failure to recognize “treatment resistance” as a diagnostic warning sign. The patient sequentially received ensartinib and lorlatinib, both of which have high intracranial activity against ALK fusion-positive lung cancer, and underwent stereotactic radiotherapy for the solitary intracranial lesion. Despite this multimodal approach, the lesion showed persistent progression, a pattern markedly inconsistent with the typical response of ALK-positive brain metastases to combined TKI and radiotherapy. While such resistance does not definitively exclude brain metastasis—given possibilities such as biological heterogeneity, acquired resistance mutations, or inadequate local control (26, 27)—it fundamentally challenges the original diagnostic assumption and should trigger multidisciplinary re-evaluation, including reassessment of imaging characteristics, treatment response, and the need for biopsy.

In this case, anchoring bias—over-reliance on the initial impression of brain metastasis—may have contributed to the interpretation of progressive neurological deficits and radiological enlargement under lorlatinib as merely “treatment failure” rather than “diagnostic inconsistency”, thereby potentially contributing to the delay in MDT consultation and subsequent definitive diagnosis. An integrated MDT discussion involving thoracic surgery, respiratory medicine, neurosurgery, radiology, and pathology would have been optimal when lesion progression was first observed on lorlatinib, as this would have facilitated earlier consideration of alternative diagnoses. In retrospect, biopsy should have been performed before stereotactic radiotherapy. It should be acknowledged that conventional MRI alone would likely have remained insufficient for definitive discrimination even with timely MDT input; however, MDT discussion would have accelerated the decision to pursue pathological confirmation through biopsy or surgical resection, potentially reducing the diagnostic delay. Nonetheless, multidisciplinary collaboration has been demonstrated to improve both survival outcomes and diagnostic and therapeutic quality in patients with lung cancer (28), and this case reinforces its value in the follow-up management of long-term survivors, particularly when treatment response deviates from expectations.

This study has several limitations. First, NGS-based molecular testing (including ALK, EGFR, and TP53) was not performed on the glioblastoma tissue, precluding definitive proof of independent clonal origins from an evolutionary perspective and limiting exploration of underlying biological mechanisms. Second, as a single case report, the findings may not be generalizable.

## 4 Conclusion

This case serves as a cautionary reminder that in the era of long-term lung cancer survival, when an intracranial lesion exhibits primary resistance to highly effective targeted therapy and local radiotherapy, the possibility of a second primary cancer should be strongly considered. Prompt initiation of MDT consultation and timely acquisition of pathological evidence are fundamental to avoiding misdiagnosis and achieving precision treatment.

## Data Availability

The original contributions presented in the study are included in the article/supplementary material. Further inquiries can be directed to the corresponding author.

## References

[1] Bray F Laversanne M Sung H Ferlay J Siegel RL Soerjomataram I . Global cancer statistics 2022: GLOBOCAN estimates of incidence and mortality worldwide for 36 cancers in 185 countries. CA Cancer J Clin. (2024) 74:229–63. doi: 10.3322/caac.21834. PMID: 38572751

[2] Siegel RL Kratzer TB Giaquinto AN Sung H Jemal A . Cancer statistics, 2025. CA Cancer J Clin. (2025) 75:10–45. doi: 10.3322/caac.21871. PMID: 39817679 PMC11745215

[3] Horn L Wang Z Wu G Poddubskaya E Mok T Reck M . Ensartinib vs Crizotinib for patients with anaplastic lymphoma kinase-positive non-small cell lung cancer: A randomized clinical trial. JAMA Oncol. (2021) 7:1617–25. doi: 10.1001/jamaoncol.2021.3523. PMID: 34473194 PMC8414368

[4] Solomon BJ Liu G Felip E Mok TSK Soo RA Mazieres J . Lorlatinib versus crizotinib in patients with advanced ALK-positive non-small cell lung cancer: 5-year outcomes from the phase III CROWN study. J Clin Oncol. (2024) 42:3400–9. doi: 10.1200/jco.24.00581. PMID: 38819031 PMC11458101

[5] Peters S Camidge R Dziadziuszko R Gadgeel S Shaw AT Kim DW . Alectinib versus crizotinib in previously untreated ALK-positive advanced non-small cell lung cancer: final overall survival analysis of the phase III ALEX study. Ann Oncol. (2026) 37:92–103. doi: 10.1016/j.annonc.2025.09.018. PMID: 41110693

[6] Uprety D Abrahami D Marcum ZA Li B Sang A Davis M . Brain metastases and mortality in patients with ALK + metastatic non-small cell lung cancer treated with second-generation ALK tyrosine kinase inhibitors as first-line targeted therapies: An observational cohort study. Lung Cancer. (2025) 201:108436. doi: 10.1016/j.lungcan.2025.108436. PMID: 39947094

[7] Liu G Abrahami D Ramachandran P Messina R Rifi N Yang H . Real-world incidence, prevalence, and clinical impact of brain metastases in patients with ALK-positive metastatic non-small cell lung cancer treated with first-line ALK tyrosine kinase inhibitors. Lung Cancer. (2026) 212:108858. doi: 10.1016/j.lungcan.2025.108858. PMID: 41500084

[8] Kishimoto T Hashimoto H Ono T Okada K . Synchronous double Malignancy: adenocarcinoma of lung and Malignant astrocytoma induced by asbestos exposure. Cancer Invest. (1992) 10:129–33. doi: 10.3109/07357909209032773. PMID: 1551022

[9] Bari KU Danish R Azher Q Karim AS . Glioblastoma multiforme in a patient with a small cell lung cancer: case report. Clin Neurol Neurosurg. (2011) 113:78–9. doi: 10.1016/j.clineuro.2010.08.014. PMID: 20889253

[10] Nukaga S Naoki K Yasuda H Kawada I Ohara K Soejima K . Secondary brain neoplasm after stereotactic radiosurgery in patients with metastatic non-small cell lung cancer. Intern Med. (2018) 57:2383–7. doi: 10.2169/internalmedicine.0184-17. PMID: 29526937 PMC6148163

[11] Qian DC Weinberg BD Neill SG Goodman AL Olson JJ Voloschin AD . Co-occurrence conundrum: Brain metastases from lung adenocarcinoma, radiation necrosis, and gliosarcoma. Case Rep Oncol. (2021) 14:487–92. doi: 10.1159/000514297. PMID: 33976625 PMC8077550

[12] Shahsavari N Ahmad M Sekar V Meola A Hancock SL Chang SD . Synchronous glioblastoma and brain metastases: illustrative case. J Neurosurg Case Lessons. (2022) 3:CASE2171. doi: 10.3171/case21714. PMID: 36273867 PMC9379681

[13] Carmicheal J Johnson KC Neilsen BK Shonka N Zhang C Baine M . Synchronous double primary Malignancy of non-small cell lung cancer and glioblastoma: A case report and literature review. Case Rep Oncol. (2025) 18:330–8. doi: 10.1159/000543770. PMID: 40881968 PMC11908813

[14] Hijab A Eran A Tzuk-Shina T Kaidar-Person O . Tumor board report: The value of tissue diagnosis. Curr Probl Cancer. (2020) 44:100530. doi: 10.1016/j.currproblcancer.2019.100530. PMID: 31771791

[15] Fordham AJ Hacherl CC Patel N Jones K Myers B Abraham M . Differentiating glioblastomas from solitary brain metastases: An update on the current literature of advanced imaging modalities. Cancers (Basel). (2021) 13:2960. doi: 10.3390/cancers13122960. PMID: 34199151 PMC8231515

[16] Peker E Ünal S Uludağ SB Zorlu NSY . Ring-enhancing lesions-differentiation with MRI. Br J Hosp Med (Lond). (2024) 85:1–20. doi: 10.12968/hmed.2024.0195. PMID: 39475033

[17] Zhang L Liu J Yang D Ni Z Lu X Liu Y . A nomogram based on consolidation tumor ratio combined with solid or micropapillary patterns for postoperative recurrence in pathological stage IA lung adenocarcinoma. Diagnostics (Basel). (2023) 13:2376. doi: 10.3390/diagnostics13142376. PMID: 37510119 PMC10378621

[18] Herba M Boczek S Smyła-Gruca W Kost K Czyżewski D Rydel M . Spread through air spaces (STAS) as a predictive and prognostic factor in patients with non-small cell lung cancer-systematic review. Cancers (Basel). (2025) 17:1696. doi: 10.3390/cancers17101696. PMID: 40427193 PMC12110037

[19] Kadota K Nitadori JI Sima CS Ujiie H Rizk NP Jones DR . Tumor spread through air spaces is an important pattern of invasion and impacts the frequency and location of recurrences after limited resection for small stage I lung adenocarcinomas. J Thorac Oncol. (2015) 10:806–14. doi: 10.1097/jto.0000000000000486. PMID: 25629637 PMC4500042

[20] Maurer MH Synowitz M Badakshi H Lohkamp LN Wüstefeld J Schäfer ML . Glioblastoma multiforme versus solitary supratentorial brain metastasis: differentiation based on morphology and magnetic resonance signal characteristics. Rofo. (2013) 185:235–40. doi: 10.1055/s-0032-1330318. PMID: 23196836

[21] Muccio CF Tedeschi E Ugga L Cuocolo R Esposito G Caranci F . Solitary cerebral metastases vs. high-grade gliomas: Usefulness of two MRI signs in the differential diagnosis. Anticancer Res. (2019) 39:4905–9. doi: 10.21873/anticanres.13677. PMID: 31519594

[22] Zhang Y Zhang H Zhang H Ouyang Y Su R Yang W . Glioblastoma and solitary brain metastasis: Differentiation by integrating demographic-MRI and deep-learning radiomics signatures. J Magn Reson Imaging. (2024) 60:909–20. doi: 10.1002/jmri.29123. PMID: 37955154

[23] Zeng Q Jia F Tang S He H Fu Y Wang X . Ensemble learning-based radiomics model for discriminating brain metastasis from glioblastoma. Eur J Radiol. (2025) 183:111900. doi: 10.1016/j.ejrad.2024.111900. PMID: 39733718

[24] Ahangari G Norioun H Ghaemi S Zali A . Artificial intelligence in glioblastoma diagnostics: Integrating MRI, histopathology, and molecular profiling. Cancer Treat Res Commun. (2025) 45:101040. doi: 10.1016/j.ctarc.2025.101040. PMID: 41308410

[25] Hu Y Gao C Wang Y Wen Z Yang C Deng H . Artificial intelligence in the task of segmentation and classification of brain metastases images: current challenges and future opportunities. Front Neurol. (2025) 16:1581422. doi: 10.3389/fneur.2025.1581422. PMID: 41064034 PMC12500441

[26] Minniti G D'Angelillo RM Scaringi C Trodella LE Clarke E Matteucci P . Fractionated stereotactic radiosurgery for patients with brain metastases. J Neuro-Oncol. (2014) 117:295–301. doi: 10.1007/s11060-014-1388-3. PMID: 24488446

[27] Gritsch D Brastianos PK . Molecular evolution of central nervous system metastasis and therapeutic implications. Trends Mol Med. (2025) 31:240–51. doi: 10.1016/j.molmed.2024.09.008. PMID: 39424530 PMC11908961

[28] de Castro G Souza FH Lima J Bernardi LP Teixeira CHA Prado GF . Does multidisciplinary team management improve clinical outcomes in NSCLC? A systematic review with meta-analysis. JTO Clin Res Rep. (2023) 4:100580. doi: 10.1016/j.jtocrr.2023.100580. PMID: 38046377 PMC10689272

